# The Social Area of the Breast: An Evolution Through Cultures and Centuries

**DOI:** 10.1093/asjof/ojae095

**Published:** 2024-10-24

**Authors:** Amitkumar M Bagdia, Nicola Rocco, Giuseppe Catanuto, Fabrizia Calenda, Ernesto Buccheri, Amedeo Villanucci, Madhusudan S Bagdia, Chaitanyanand Koppiker, Maurizio Bruno Nava

## Abstract

Breasts have evolved as a functional and aesthetic unit. The perception of society toward social exposure of the breast has been extremely diverse, not only temporally through the same civilization but also among contemporary parallel societies in geographically diverse areas. This article explores the spatial and temporal evolution of the outlook toward social exposure of breast over the past 3000 years among different societies, countries, and religions. The level of “acceptance” or “tolerance” toward visibility of breast in social situations was historically guided by social conditioning. Recently, the personal attitudes and preferences of the women themselves have become more deterministic of the clothing choices. Consequently, the conventional oncoplastic notion of a “no-scar zone” being the upper-inner quadrant of the breast must also be redefined to match the dynamic personal preferences of the patient. It is important to know what the patient thinks is the “most social” area of the breast for her, and then to avoid scars in those areas if possible. The article discusses the quest to minimize scars and the importance of shared decision making.

## EVOLUTIONARY BIOLOGY

Over 300 million years ago, Mammalia differentiated themselves from the Reptiles and Birds of the Carboniferous period by the presence of mammary glands to enable feeding the offspring. Eutherian mammals started to display sexual dimorphism with only the women having functional breasts. Overtime, breasts also acquired the role of secondary sexual organ: assisting reproduction without directly being involved in gametogenesis. A woman displaying healthy breast capable of feeding the progeny was viewed as a desired sexual partner by the males. This evolutionary mechanism of signaling woman sexual competence by mammary display has continued to attract male attention.

## SOCIAL EVOLUTION

Each society had its own level of acceptance toward body exposure. Spectrum varied from ancient Greco-Roman cities with socially bare breasts to the Islamic settlements in Saudi where minutest of exposed skin was sacrilegious.

In many ancient cultures, females' clothing was often quite revealing, with little to no coverage of the breasts. This was especially true where women were considered to be sacred or divine. For example, in ancient Egypt, women were often depicted wearing only a sheer veil or a loincloth. In ancient Greece, women often wore a chiton, which was a simple garment that left the breasts exposed. Some Greek philosophers theorized that women should be topless to promote sexual equality. Some advocated that breasts are not private or sexual parts at all. Over time, as cultures became more patriarchal, females' clothing began to cover more of the body.

Ancient Indian civilizations often had women being topless, but this changed subsequently after Mughal invasion, leading women to cover-up to protect against intrusive male gaze, saving their modesty and objectification. The general presumption that the Western World has always been liberal and the Eastern/Indic societies have been more restrictive in their acceptance of social breast exposure is not factually accurate (Tables 1 and 2).

**Table 1.** Socially Acceptable Breast Exposure in the Western World Through the Centuries

Era	Socially acceptable breast exposure
Ancient Times	Breast exposure equated as symbol of fertility and beauty, with Greek and Roman clothing often featuring draped garments that left the breasts partially or fully exposed
Medieval Era	Christian influence led to a shift toward modesty and concealment of the female body. Females' clothing covered the breasts entirely, with high necklines and tightly fitted bodices
Renaissance	Renaissance art celebrated the female form, afforded slight relaxation in societal norms, allowing for lower necklines and the breast-enhancing corsets
18th century	Rococo fashion introduced décolletage, with low-cut dresses and gowns, revealing the upper part of the breasts. Cleavage was emphasized and was embraced by aristocratic women
Victorian Era	Strict social standards and a focus on modesty ensued. High necklines, corsets, and layers of undergarments covered the breasts entirely
Early 20th century	Clothing standards were relaxed again. The “Flapper” females' short skirts and looser silhouettes were popular, but breasts remained largely covered
Mid-20th century	Introduction of the Bikini normalized circumstantial breast exposure. With a push for gender equality, women embraced braless fashion and low-cut tops
Late 20th century	Significant breast exposure was normalized. Plunging necklines, sheer fabrics, and open-back clothing were commonplace

**Table 2.** Socially Acceptable Breast Exposure in India Through the Centuries

Era	Socially acceptable breast exposure
Ancient Times	Clothing was often quite revealing. In the Indus Valley Civilization, women often wore a simple loincloth or skirt. In the Vedic period, women wore a long tunic called a sari. These would occasionally leave the breast uncovered
Medieval Era	With the spread of Islamic influence, societal norms became more prohibitive. Breasts were covered entirely. Sari began to be paired with a blouse that covered the breasts
Mughal Empire	Fitted bodices and translucent fabrics in garments, such as the Anarkali and Choli, accentuated the shape of the breasts while still providing coverage
Colonial Era	Under the British, Victorian ideals of modesty were imposed, leading to the covering of the female body, including the breasts, with blouses and high-necked clothing
Indian Independence	Following India's independence in 1947, traditional clothing like sari and salwar kameez were reviewed. These garments provided modest coverage of the breasts, with customizable draping styles and necklines based on regional and cultural preferences
Modern Era	Notably increasing influence of global fashion trends. Western-inspired clothing with lower necklines, cutouts, and sheer fabrics has gained popularity. However, societal norms and regional/local cultural factors still play a significant role in determining acceptable levels of breast exposure

In most contemporary societies, clothing choice is driven by personal preference. Although taboos and civil codes exist, most are directory rather than mandatory in nature. Women are increasingly choosing to wear clothing that makes them feel confident and comfortable, while also being able to express their identity and culture. Modern Indian women in Sari or Mexican women in Huipil reflect the contemporary sociocultural and personal preferences. Contrarily, some Islamic cultures continue to have strict dressing codes, including a hijab, niqab, or burqa (Tables 3 and 4).

**Table 3.** Socially Acceptable Breast Exposure in Different Religions

Religion	Socially acceptable breast exposure
Christianity	Modesty is generally emphasized. Blouses, dresses, or tops with higher necklines provide full breast coverage
Islam	Modesty is practiced and also often enforced. Modest clothing, loose-fitting tops, or dresses with high necklines and long sleeves, gear like Hijabs, burqas, and niqabs are also worn by some Muslim women
Hinduism	Traditional attire, such as the sari or salwar kameez, is commonly worn by Hindu women. These garments generally cover the breasts and provide modesty. Styles differ based on regional and cultural preferences
Buddhism	Buddhism does not prescribe specific dress codes. Modesty and cultural norms play a significant role. Breasts are covered. Buddhist nuns wear robes that cover the entire body
Judaism	In Orthodox Judaism, modesty is highly valued, breasts are covered entirely. Conservative and Reform Jewish communities may have more diverse interpretations, valuing modesty
Sikhism	Sikhism promotes modesty and dignified appearance. Sikh women traditionally wear the Punjabi suit, which includes a long tunic (kameez) and pants (salwar). These garments typically cover the breasts and provide modesty

**Table 4.** Socially Acceptable Breast Exposure in Different Countries

Country	Socially acceptable breast exposure
United States	Modesty is generally valued. Females' clothing typically covers the breasts entirely, with higher necklines and conservative styles
Europe	Europe tends to have a more relaxed attitude toward breast exposure. Low-cut necklines, revealing swimwear, and occasional glimpses of cleavage are more accepted, especially in fashion-forward urban areas
Japan	Japanese culture generally emphasizes modesty and discretion. Females' clothing in Japan often covers the breasts entirely, with high necklines and conservative styles. Traditional attire like the kimono provides full coverage
Brazil	Brazil has a more accepting attitude toward breast exposure, particularly in beach culture. Swimwear styles, such as bikinis and 1 pieces with plunging necklines, are common and widely accepted. However, in other settings, modesty is still expected and higher necklines may be preferred
Saudi Arabia	Saudi Arabia follows strict Islamic principles that prioritize modesty. Females' clothing must cover the breasts entirely, with high necklines, loose-fitting styles, and typically long sleeves. Abayas and niqabs are commonly worn to ensure full coverage
India	India has diverse cultural practices, and acceptable breast exposure can vary. Traditional attire like the sari or salwar kameez generally covers the breasts, with different draping and necklines based on regional and cultural preferences. Modesty is valued
Australia	Australia tends to have a more relaxed approach to breast exposure. Bikinis and 1 pieces with various necklines, including lower cut options are prevalent in appropriate settings
United Kingdom	Conservative breast coverage is the norm in most public settings. Low-cut necklines may be acceptable in certain social and formal occasions

## “SOCIAL AREA” OF THE BREAST

It is evident that there is no “single best and universally applicable” definition of “social area” of the breast. Further, with the introduction of plethora of modern dressing trends and fashions, many different “types of cleavages” have evolved, like the vernacularly called side boob or the under boob (Table 5). Social area of the breast is now a dynamic and personal preference rather than a rigid uniformly defined surgical territory of the upper-inner quadrant (UIQ) of the breast. The contemporary dogmatic oncoplastic consensus regarding the UIQ universally being the “no-man's land” or the “no-scar zone” because of being the social area is only a slice of the entire truth. Hence, before placing any incision, it is imperative for any surgeon to take into consideration the patients’ perception on what is the “most social” area of the breast “in her own opinion” and accordingly avoid a scar in that zone, if oncologically feasible.

**Table 5.** Breast Exposure According to Different Clothing Styles Though Different Cultures

Exposed region of the breast	Clothing styles
Upper breast exposure	Low cut/plunging necklines, off-shoulder dresses
Lower breast exposure	Underbust, certain bandeaus
Inner breast exposure	Keyhole cutouts, sheer/mesh panels
Outer breast exposure	Side cutout/side boobs
Nipple–areola exposure	Sheer/transparent styles, cutouts, string bikinis

## IMPLICATIONS FOR THE ONCOPLASTIC SURGEONS

The rapidly evolving social scenario and also the variability of customs world-over have made it imperative to be mindful of patients personal choices (“What is the most social area of the breast in her own opinion”) when it comes to placing an incision on the breast. The classical oncoplastic teaching of designating the UIQ as the default “no-scar” zone is no longer appropriate. Similarly, the classical notion of certain zones of the breast being “safe” or preferred scar placement zones, like the infra-mammary crease, lateral crease, or the peri-areolar area, should not be the *default* practice either. Incisions need to be chosen after a thorough discussion with the patient about the options available to her, and the pros and cons of each.

Patients’ values and preferences should be properly evaluated when taking every negotiable choice in oncoplastic breast surgery. Many times patients are not able to elicit their preferences; this is why surgeons should be trained in “shared decision making” and should acquire proper communication skills apart from surgical technical ones. A very useful approach to shared decision making is the one described by Glynn Elwyn, the so-called 3-talk model, in which the patients and the physician first create a team (“team talk”), then move to a detailed presentation of all possible therapeutic options with an evidence-based approach (“option talk”) and last go toward a final decision in which the patient's values and preferences are duly considered (“decision talk”).^1^

Various Level 1 and Level 2 oncoplastic techniques have been described in the literature to suit various specific tumor locations, patients’ and surgeon's preferences. The techniques have been elucidated quadrant wise by various authors and the ones elucidated by Clough et al have been particularly adopted widely.^2^

The plethora of available options and the absence of consensus on any of the available options being the “best” is a testament to the fact that the placement of scar is an open question, the answer to which cannot be generalized. The answer lies in the domain of “personalized medicine,” in this case, under “personalized surgery” and must be dealt with as such.

In the era of globalization, breast surgeons should increase their awareness toward possible differences among cultures and populations in taking some choices that could appear to be obliged or obvious for a surgeon working in an established cultural setting.

## A POSSIBLE FUTURE: SCARLESS SURGERY

Many patients, even celebrity women have embraced the inevitability of the scars and have chosen to embrace them. Some have chosen to even flaunt them and display them as their souvenir badge of victory over cancer so as to motivate and educate women about breast cancer.

A scar is a legacy that a surgeon leaves with his patients. We might perhaps never be able to eradicate a scar totally. Modern techniques like minimally invasive breast surgery and robotic surgeries are being investigated. Just like an endoscopic transoral thyroid surgeon has concealed the visible neck scar inside the hidden area of oral cavity, or just like the NOTES (Natural Orifice Transluminal Endoscopic Surgery) practitioner has virtually eliminated external scar, can the breast oncosurgeon safely displace the scar to the back, axilla, or another less conspicuous area? We do not know yet. Ductoscopic surgery is an advance in this direction. Besides, we have already brought the scar length down to a puncture in some cases (eg, vacuum-assisted excisions). Science is investigating a future where surgery may totally be avoided with the advent of modern chemotherapy and radiation/ablative modalities.

Surgical science has been evolving rapidly over the past few decades. The journey from dogmatic adherence to the Halstedean mastectomy to the era of nipple sparing mastectomies and breast-conserving surgeries has been the result of incremental advancements and occasional leaps in our understanding of breast cancer biology. The future looks brighter than ever, and hopefully, the last chapter in the book of Oncoplasty has not been written yet.
